# Nitrile rubber biodegradation by *Gordonia* sp. strain J1A and discovery of an oxygenase involved in its degradation

**DOI:** 10.1128/aem.02128-25

**Published:** 2025-11-26

**Authors:** Takehiro Chiba, Takuma Nakaarai, Daisuke Sugimori

**Affiliations:** 1 Faculty of Symbiotic Systems Science and Technology, Fukushima University 12776, Fukushima, Japan; University of Milano-Bicocca, Milan, Italy

**Keywords:** nitrile rubber, biodegradation, enzymes, environmental microbiology

## Abstract

Acrylonitrile‐butadiene rubber (NBR), which is a petroleum-derived synthetic copolymer composed of acrylonitrile and 1,3-butadiene, is used in large amounts around the world for disposable gloves and oil seals. Most of which are incinerated without being reused. We aimed to develop a bio-upcycling method for waste NBR (wNBR) and studied microorganisms capable of assimilating NBR. An NBR-degrading actinomycete, *Gordonia* sp. strain J1A, which degraded 6.5%–11% in weight loss of a wNBR sample in 10 days, was isolated from an activated sludge of an industrial wastewater treatment plant. We have found a membrane-bound nitrile rubber oxygenase (Nro1) as a key enzyme that catalyzes the initial reaction step of NBR decomposition. Nro1 showed high homology to the amino acid sequences of MpaB family proteins, but no similarity to those of natural rubber-degrading enzymes (latex clearing proteins: Lcps). The degradation products, such as 4-cyano-1-cyclohexene and C22-C58 NBR oligomers, containing aldehyde or ester, as well as carbon black, were detected through the enzymatic degradation of the wNBR sample by Nro1. These findings offer a new strategy for a bio-upcycling method of wNBR.

**IMPORTANCE:**

An actinomycete, *Gordonia* sp., utilized multiple acrylonitrile‐butadiene rubber (NBR)-degrading enzymes to cleave the main-chain carbon–carbon (C–C and C=C) bonds of NBR, a petroleum-derived synthetic rubber. The degradation products, such as 4-cyano-1-cyclohexene and C22-C58 NBR oligomers, containing aldehyde or ester, were detected through the enzymatic degradation of a waste NBR (wNBR) sample. One of the NBR-degrading enzymes, nitrile rubber oxygenase (Nro1), showed high homology to the amino acid sequences of MpaB family proteins. This study provides new insights into the enzymatic degradation of the petroleum-derived synthetic polymers.

## INTRODUCTION

**Fig 1:**
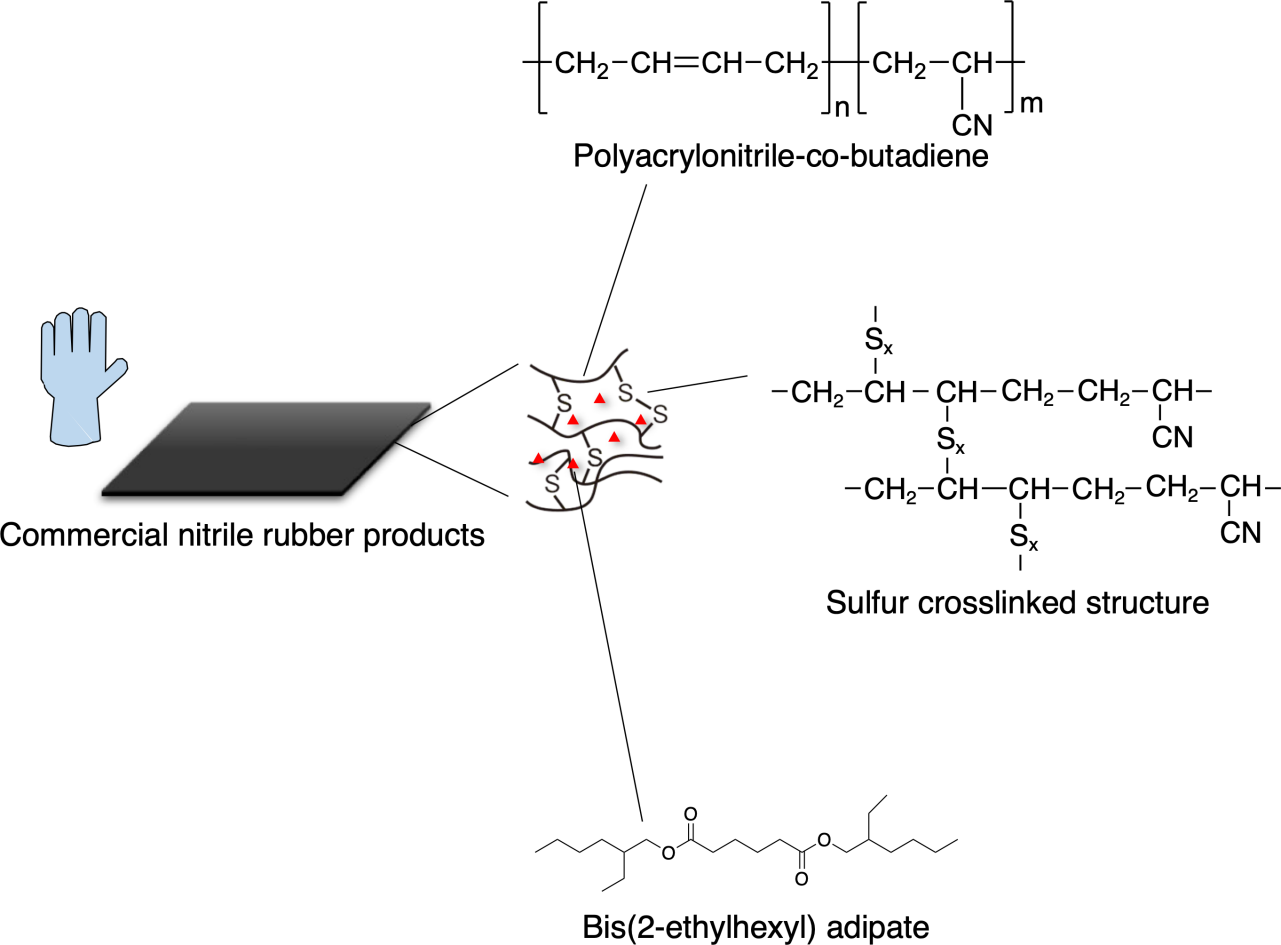
Chemical structures of compounds contained in nitrile rubber products.

Acrylonitrile-butadiene rubber, so-called nitrile rubber (NBR), which is a copolymer of acrylonitrile (AN) and 1,3-butadiene (BD), is widely used in large amounts around the world as disposable gloves and oil seals (Fig. 1). These waste materials, as well as product manufacturing by-products and offcuts, are discarded in large quantities, most of which are incinerated without being effectively reused. For example, an oil seal manufacturer in Japan discharges at least 10 kt/yr NBR as offcuts, which is almost equal in weight to the products. The amount of waste NBR (wNBR) emitted in Japan is estimated to be at least 0.1 Mt/yr according to the Japan Rubber Manufacturers Association (https://www.rubber.or.jp/page3.html?id=6). The amount of NBR produced globally in 2020 was at least 1.32 Mt/yr and is expected to increase according to The International Institute of Synthetic Rubber Producers, Inc. (https://iisrp.com/). Therefore, it is needed to develop an effective recycling method for wNBR. If NBR-assimilating microorganisms could be obtained, the enzymes involved in NBR metabolism would be valuable for exploiting a new upcycling method for wNBR. Recently, Delgado-Nungaray et al. reported that the Gram-negative bacterium, *Pseudomonas aeruginosa,* degraded 1.66% of NBR glove in liquid culture (1). However, to the best of our knowledge, there has not been any report on NBR degradation enzymes and the assimilation pathway.

Here, we report the isolation of an actinomycete, *Gordonia* sp. strain J1A, capable of degrading wNBR emitted from an oil seal manufacturing factory. In general, NBR products contain sulfur crosslinked (sc) structures and various additives, such as plasticizers, anti-aging agents, and antioxidants. Our findings suggested that strain J1A can degrade not only the sc structures and a plasticizer but also the main-chain carbon–carbon (C–C and C=C) bonds of NBR. We also detected 4-cyano-1-cyclohexene and C22-C58 NBR oligomers containing aldehyde, ester, and hydroxyl groups as the degradation products. In addition, we identified a membrane-bound nitrile rubber oxygenase (Nro1) and its coding gene from strain J1A. Nro1 showed high amino acid sequence similarity to MpaB family proteins (2). These results indicate that Nro1 cleaves the main-chain C=C bond in the poly(acrylonitrile-butadiene) component of NBR, in a manner similar to the oxidative attack catalyzed by MpaB family proteins (2) and the known latex clearing protein (Lcp) (3, 4).

## MATERIALS AND METHODS

### NBR samples and chemicals

The NBR samples were provided by NOK Corporation (Kanagawa, Japan). The wNBR sample was discharged from NOK Corporation’s oil seal manufacturing factory. The scNBR sample contained the sc structure without any other additives, such as plasticizers, anti-aging agents, and antioxidants. The pNBR sample contained no sc structure or additives. The scNBR and pNBR samples were synthesized by the NOK Corporation. BMtouch (powder-free), as a commercial latex glove was obtained from BMBio Co. Ltd. (Tokyo, Japan). *cis*-1,4-Polyisoprene (IR) and polyacrylonitrile (PAN) were purchased from Sigma-Aldrich. Bis(2-ethylhexyl) adipate (DOA), presumed as a plasticizer, was obtained from Tokyo Chemical Industry Co. Ltd. (Tokyo, Japan).

### Screening

Soil samples were collected from within Fukushima Prefecture and suspended in distilled water. Wastewater and activated sludge samples from an industrial wastewater treatment plant were collected from NOK Corporation’s factory. The soil sample suspension or wastewater (50 µL) was transferred to a test tube (⌀18 × 180 mm) containing 5 mL of W medium (1.0 g of KH_2_PO_4_, 8.0 g of K_2_HPO_4_, 0.1 g of NaCl, 50 mg of yeast extract, 200 mg of MgSO_4_·7H_2_O, 20 mg of CaCl_2_·2H_2_O, 18.3 mg of FeSO_4_·7H_2_O, 0.5 mg of Na_2_MoO_4_·2H_2_O, 0.5 mg of Na_2_WO_4_·2H_2_O, and 0.8 mg of MnSO_4_·5H_2_O per liter) with 5 mg of wNBR sample (ca. 10 × 7 × 2 mm) as a carbon and nitrogen source. The test tube was shaken at 160 strokes/min at 28°C. The samples (1%) where the cell growth was observed as turbidity were transferred to the 5 mL fresh W medium with the wNBR sample and incubated under the same conditions. After at least three successive transfers, a single colony was isolated on nutrient agar, and the degradation rate of the isolates was determined by measuring the weight loss of the wNBR sample as described below. Strain J1A, showing the highest degradation rate, was selected for further study and taxonomically identified by full-length 16S rDNA analysis (TechnoSuruga Laboratory Co., Ltd., Shizuoka, Japan).

### NBR degradation assay

The NBR sample after the cultivation was immediately washed three times in distilled water and then dried in a vacuum desiccator until it reached a constant weight. The dry weight of the NBR sample was measured. NBR degradation rate was calculated based on the weight loss by comparing to the control sample without bacterial inoculation.

For enzymatic degradation, one piece of wNBR or pNBR sample (~300 mg) was added to 5 mL of enzymatic reaction solution and incubated at 160 strokes/min for 24 h at 37°C. After the incubation, the dry weight of the NBR sample was measured by the same method described above. NBR degradation rate was calculated based on the weight loss by comparing with the control sample inactivated by using 15% trichloroacetic acid.

### Biodegradation of NBR, other rubbers, and additives by strain J1A

The growth of strain J1A in the W medium containing IR, PAN, DOA, or a 1.0 × 1.5 cm piece of a commercial latex glove, BMtouch (BM Equipment Co., Ltd., Tokyo, Japan). The cell growth was determined by colony-forming units on a nutrient agar plate.

### Purification of the NBR-degrading enzymes

Strain J1A was grown on an agar plate containing 0.75% tryptic soy broth (TSB; Becton Dickinson) for 1 day at 37°C. A single colony on the plate was inoculated into the test tube containing 5 mL of 0.75% TSB medium with one piece of wNBR sample (ca. 10 × 7 × 2 mm, ca. 5 mg), and shaken at 160 strokes/min for 1 day at 37°C. Each culture (1 mL) was inoculated into 100 mL of 0.75% TSB medium in a 500 mL baffled flask containing five pieces of wNBR sample (ca. 10 × 10 × 10 mm, ~60 mg), wNBR, and incubated for 1 day at 37°C. The cells were harvested by centrifugation (18,000 × *g*, 30 min, 4°C), and the culture supernatant (sup) was collected. The cell paste (1 g of wet cells) was washed and resuspended in 10 mL of 20 mM Tris-HCl, pH 8.0 (A buff.). The cell suspension was sonicated at 50 kHz (TP-040, Tomy Seiko, Tokyo, Japan) on ice. The lysate was then centrifuged (18,000 × *g*, 30 min, 4°C), and the supernatants were obtained as cell-free extracts (cfe), and then precipitants (ppt) were collected and resuspended in 5 mL of A buff. Likewise, the lysate (100 mL) prepared with 10 g of wet cells was solubilized in 0.7% 3-[(3-cholamidopropyl)dimethylammonio]-1-propanesulfonate (CHAPS) while stirring gently for 17 h at 4°C. The solubilized lysate fraction was centrifuged (18,000 × *g*, 30 min, 4°C), and the supernatant was collected. The supernatant was loaded at a rate of 5 mL/min into an anion exchange chromatography column (Toyopearl DEAE-650M, Tosoh, Tokyo, Japan) that had been equilibrated with A buff. Three-column volumes (CVs) of A buff. were used for washing. Elution was carried out with a linear gradient (10 CV) of an elution buffer (1 M NaCl/A buff.). The NBR degradation activity of each fraction was determined by measuring the weight loss of wNBR and pNBR samples.

### Scanning electron microscopy

One piece of the wNBR sample (ca. 300 mg) was incubated with 5 mL of sup, ppt fraction, and buffer control sample at 160 strokes/min for 24 h at 37°C and washed and dried as described above. The wNBR samples were examined using a scanning electron microscope (SEM) (SU3500, Hitachi) at 15 kV.

### Field emission electron probe microanalyzer analysis

The wNBR samples were also analyzed using a JXA-8500F system (JEOL). The analytical conditions were as follows: the acceleration voltage was 10 kV; the irradiation current was 100 nA; the analysis area was 3,500 μm × 3,500 µm; the measurement time was 50 mS/point; and the pixel number was 250 × 250 points. The X-ray analysis was conducted using the following crystals: C Kα (LDE2H), N Kα (LDE5H), and O Kα (LDE1).

### Carbon-13 nuclear magnetic resonance spectroscopy

The active fraction of the DEAE chromatography column (DEAE-purified sample) and the buffer control sample were allowed to react with the wNBR sample at 160 strokes/min for 24 h at 37°C, and then the wNBR sample was washed and dried as described above. Those NBR samples and a virgin NBR sample were swollen in CDCl_3_ solution and then analyzed by carbon-13 nuclear magnetic resonance (^13^C-NMR) (ECX-400, JEOL) at 60°C using a gated decoupling method with 12-s waiting time and 6,500 scans. The contents of BD and AN units were estimated based on the ratio of the intensities of the signals attributable to C=C double bonds (125–135 ppm) and cyano (CN) groups (115–125 ppm).

### Gas chromatography–mass spectrometry

The ppt fraction and the buffer control sample were allowed to react with the pNBR sample in a septa-sealed glass vial (⌀22 × 32 mm) at 160 strokes/min for 24 h at 37°C. After the enzymatic reaction, the gas phase was analyzed by gas chromatography–mass spectrometry (GC–MS) (GC: GC-2030, MS: GCMS-TQ8050NX, Shimadzu) equipped with an InertCap Pure-WAX column (GL Sciences).

### Matrix-assisted laser desorption ionization-time-of-flight mass spectrometry

The ppt fraction and the buffer control sample were allowed to react with the pNBR sample at 160 strokes/min for 24 h at 37°C. After the enzymatic reaction, the liquid phase was mixed in equal volume with a matrix solution comprised of 20 mg/mL dithranol and 1 mg/mL silver trifluoroacetate in tetrahydrofuran and then analyzed by matrix-assisted laser desorption ionization-time-of-flight mass spectrometry (MALDI-TOF-MS) (rapifleX, Bruker).

### Fourier-transform infrared spectroscopy

The wNBR and pNBR samples were incubated with 5 mL of the DEAE-purified sample and the buffer control sample at 160 strokes/min for 24 h at 37°C, respectively. After the enzymatic reaction, the white precipitants were produced from both samples and then collected by centrifugation (9,900 × *g*, 10 min, 4°C). The obtained precipitants were dried under vacuum and mixed with KBr powder to prepare the pellet, and then analyzed by Fourier-transform infrared (FT-IR) spectroscopy (FT/IR-4100, Jasco).

### Whole genome sequence analysis

Genomic DNA was extracted from strain J1A cell pellet, which was cultivated in 0.75% TSB medium with 300 mg of the wNBR sample for 24 h at 37°C, by a genomic-tip 100 G (Qiagen). Strain J1A’s whole genome was analyzed by short-read and long-read sequencing. For short-read sequencing, the DNA library was prepared using an MGIEasy FS DNA Library Prep Set (fragmentation performed for 4 min at 32°C), an MGIEasy DNA Adapters-96 (Plate) Kit, an MGIEasy Circularization Kit, and a DNBSEQ-G400RS High-throughput Sequencing Kit, according to the manufacturers’ protocols. The sequencing step was carried out using DNBSEQ-G400, 2 × 200 bp (all kits were supplied by MGI Tech Co., Ltd., China). Cutadapt (ver. 2.7) was used to remove the adapter sequences, Seqkit (ver. 0.11.0) to sample the read sequences, and Sickle (ver. 1.33) to trim low-quality reads (quality threshold: 20; length threshold: 147). The DNA library for long-read sequencing was prepared using a Ligation Sequence Kit. Sequence data were acquired by GridION using an R9.4.1 flow cell (Oxford Nanopore Technologies, UK). *Guppy* (ver. 4.0.11 + f1071ce) was used for the base calling and barcoding of data. Porechop (ver. 0.2.3) was used to remove adapter sequences, and Filtlong (ver. 0.2.0) discarded reads shorter than 1 kb. Finally, sequences acquired by short and long read sequencing were assembled by Unicycler (ver. 0.4.7). The quality of the assembled genome was checked using Bandage (ver. 0.8.1) and CheckM (ver. 1.1.2). The coding sequences were predicted by Prokka (ver. 1.14.5) and DFAST (https://dfast.ddbj.nig.ac.jp/).

### RNA-seq analysis

Strain J1A was cultivated with and without the wNBR sample using the same method described above. Strain J1A cell pellet was suspended in Buffer RLT, including 2-mercaptoethanol (Qiagen). The total RNA was extracted using an RNeasy Mini Kit (Qiagen). rRNAs were removed using riboPool (siTOOLs Biotech, Germany), and the cDNA library was prepared using an MGIEasy RNA Directional Library Prep Set, an MGIEasy Circularization Kit, and a DNBSEQ-G400RS High-throughput Sequencing Kit, according to the manufacturer’s protocols. The sequencing step was carried out using DNBSEQ-G400, 2 × 100 bp (all kits and manufacturer’s protocols were supplied by MGI Tech). The acquired data were mapped to the assembled strain J1A whole genome sequence. The mapped reads were counted using featureCounts (ver. 2.0.0) and normalized by RPKM (reads per kilobase of exon per million mapped reads). The inducted genes were identified by comparing RPKM values with and without the wNBR sample.

### Production of recombinant Nro1

The recombinant Nro1 was produced in *Escherichia coli* C43 (DE3) containing pET24a-*nro1*-6×His. The recombinant cells were grown in LB medium (10 g of tryptone, 5 g of yeast extract, 10 g of NaCl, 50 mg of kanamycin per liter, pH 7.0) at 160 strokes/min for 18 h at 37°C. The cell cultures (1 mL) were inoculated into 100 mL of TB medium (12 g of tryptone, 24 g of yeast extract, 5 g of glycerol, 2.3 g of KH_2_PO_4_, 12.5 g of K_2_HPO_4_, 50 mg of kanamycin per liter) in a 500 mL baffled flask. They were then incubated at 160 strokes/min for 3 h at 37°C, and subsequently incubated with 0.5 mM isopropyl β-d-1-thiogalactopyranoside for 4 h at 30°C. The recombinant cells were harvested by centrifugation (18,000 × *g*, 30 min, 4°C) and disrupted by sonication after resuspension in 20 mM Tris-HCl buffer (pH 8.0). To assay NBR degradation activity, the cell lysate was incubated with ca. 250 mg of wNBR or pNBR at 160 strokes/min for 24 h at 37°C. After the incubation, the NBR degradation rate was determined by the same method described above.

Aldehyde/ketone of the reaction products was detected by 2,4-dinitrophenylhydrazine hydrochloride (2,4-DNPH) (5). The reaction mixture (0.4 mL) after the enzymatic reaction was mixed with 0.4 mL of 1 mg/mL 2,4-DNPH/methanol and 0.2 mL of 1 M HCl and followed by incubation at 40°C for 10 min, and then added 0.2 mL of 8 M NaOH. After centrifugation (21,600 × *g*, 5 min, 4°C), the absorbance of the supernatant (100 µL) was measured at 450 nm. The concentration of aldehyde/ketone was determined based on the calibration curve with 0 to 10 mM hexanal as a standard.

Oxygen consumption rate was measured using an oxygen electrode (FireStingO_2_-C; BAS Inc., Tokyo, Japan) (4). One piece of wNBR or pNBR sample (ca. 20 mg) was added to 2 mL of cfe of recombinant cells in ⌀18 × 180 mm test tube and incubated at 37°C. The oxygen electrode was inserted to a depth of 0.5 cm from the reaction mixture surface, and the oxygen concentration was measured.

## RESULTS AND DISCUSSION

**Fig 2:**
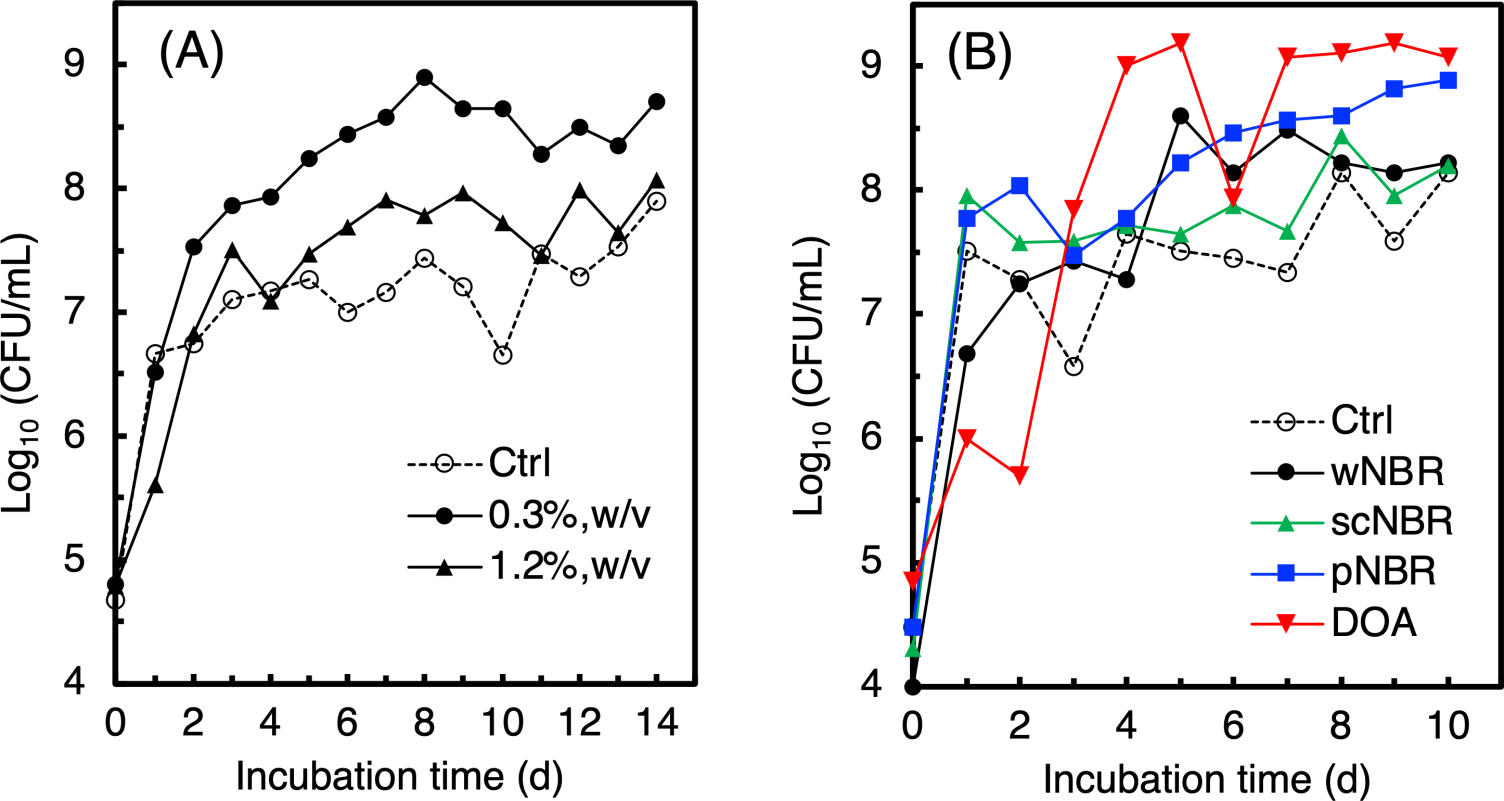
Effects of wNBR content (**A**), rubber composition, and additives (**B**) on cell growth in W medium. DOA, bi(2-ethylhexyl) adipate expected as a plasticizer added to wNBR; Ctrl, NBR-free control (**A**) or w, sc, pNBR- and DOA-free control (**B**).

A wNBR-assimilating actinomycete, *Gordonia* sp. strain J1A, has been isolated from the activated sludge sample of the wastewater treatment in the oil seal manufacturing factory (Fig. 2A). The 16*S r*DNA analysis showed that strain J1A was assigned to be a close relative of *Gordonia cholesterolivorans*. The 6.5%–11% weight loss of the wNBR sample caused by strain J1A during the biodegradation after 10 days at 37°C in the W minimal medium. Moreover, the results of the growth curve analysis suggested that strain J1A may degrade pNBR and scNBR, as well as DOA (Fig. 2B). Although it exhibited poor growth in the W medium with the commercial latex glove and IR, there was no growth with PAN (data not shown). Although IR is all *cis* form, the latex glove contains the *trans* form. Hence, we thought that strain J1A can degrade *trans* form, but not *cis* form. In addition, an excessive content of wNBR (>1.2%, wt/vol) inhibited the cell growth (Fig. 2A), suggesting that the growth might be inhibited by the additives in wNBR. Recently, Delgado-Nungaray et al. reported that *P. aeruginosa* degraded 1.66% of NBR glove in liquid culture after 7 days (1). Thus, the NBR degradation ability of strain J1A was superior to that of *P. aeruginosa*.

A localization study of the enzymes involved in wNBR degradation revealed that an enzyme responsible for cleaving the main-chain C–C and C=C bonds in the poly(acrylonitrile-butadiene) backbone of NBR was located in the cell membrane (Fig. S1). A sc bond-degrading enzyme seemed to exist in the culture supernatant and the cytoplasm. An esterase capable of hydrolyzing the additives, such as DOA, appeared to exist in the culture supernatant, cell membrane, and/or cytoplasm. The results of the genome sequence and RNA-seq analyses of strain J1A supported the presence of esterases capable of hydrolyzing DOA and thiol-disulfide oxidoreductases capable of cleaving the sc bond. In fact, the most significant increases in the transcription levels of esterase and thiol-disulfide oxidoreductase genes were 94.5- and 3.1-times higher than compared with the culture in the presence of the wNBR sample, respectively (Table 1).

**TABLE 1:** Up-regulated genes in RNA-seq analysis

Rank	Locus tag	Gene name	Gene annotation	Up-regulated rate
1	J1A_05760	–^*a*^	NADH dehydrogenase-like protein	139.5
2	J1A_14580	–	Putative endopeptidase	107.0
3	J1A_21290	–	Putative GMC-type oxidoreductase	102.7
4	J1A_04870	–	Serine hydrolase domain-containing protein	94.5
5	J1A_14550	pimB	GDP-mannose-dependent alpha -(1-6)-phosphatidylinositol monomannoside mannosyltransferase	82.6
6	J1A_07590	desA1	Putative acyl-[acyl-carrier-protein] desaturase	81.3
7	J1A_14560	–	Hypothetical protein	80.4
8	J1A_07600	cobA	Uroporphyrinogen-III C-methyltransferase	70.7
9	J1A_14570	ripC	Peptidoglycan hydrolase RipC	68.0
10	J1A_25300	–	Universal stress protein	66.6
292	J1A_34400	–	MpaB family protein (nitrile rubber oxygenase, nro1 in this study)	7.0
1095	J1A_26600	resA	Thiol-disulfide oxidoreductase	3.1

^*a*^ –, not applicable.

**Fig 3:**
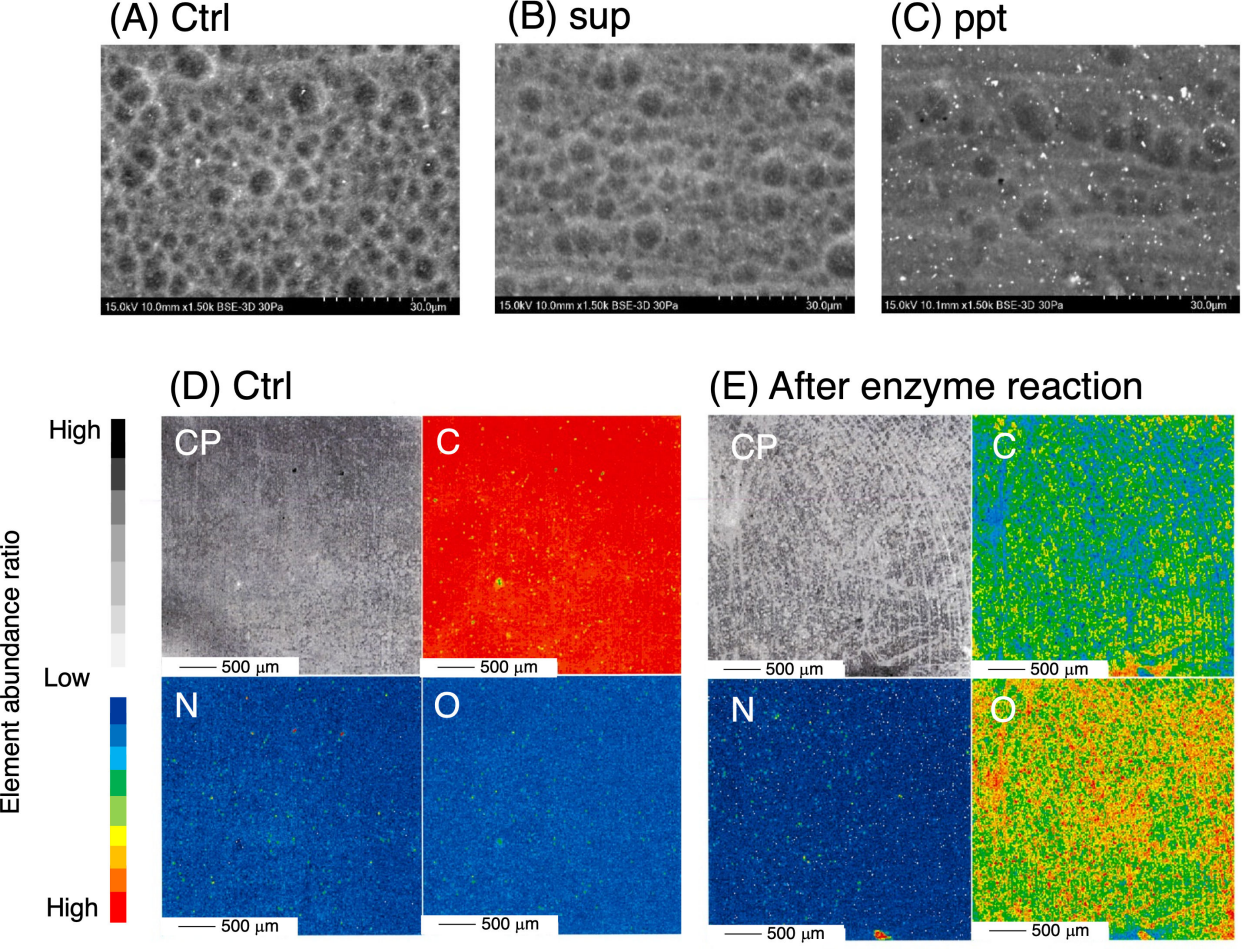
SEM (**A–C**) and FE-EPMA mapping images (**D and E**) of wNBR surface after enzymatic degradation (37°C, 24 h). Ctrl, after incubation with only 20 mM Tris-HCl buffer (pH 8.0); sup, culture supernatant; ppt, cell membrane fraction; CP, compositional image; C, carbon atom; N, nitrogen atom; O, oxygen atom.

The partially purified enzyme sample degraded 5.3 mg of wNBR (degradation rate, 1.7%) and 1.8 mg of pNBR (degradation rate, 0.58%) at 37°C and pH 8.0 in 24 h. SEM and field emission electron probe microanalyzer (FE-EPMA) analyses showed that the wNBR surface was shaved by the enzymatic reaction (Fig. 3A through C). Additionally, the ppt fraction appeared to be more active than the sup sample because of the fewer bubbles. FE-EPMA analysis also demonstrated that the contents of carbon and nitrogen atoms decreased by 37% and 7.4%, respectively (Fig. 3D and E). Although the oxygen atom content increased by 2.4 times compared with the control wNBR sample, no groups containing oxygen atoms, such as carbonyl groups (C=O) at 170–180 ppm, were detected by ^13^C-NMR analysis (Fig. S2). From these results, we concluded that the increase in the oxygen atom content was probably due to exposure to SiO_2_ and/or CaCO_3_ fillers.

The enzyme degraded pNBR with an AN content of 40% approximately three times faster than that with an AN content of 28%. ^13^C-NMR analysis demonstrated that the AN unit content of wNBR was reduced from 39 wt% to 34 wt% by the enzymatic degradation (Table 2). Consequently, the molar ratio of BD to AN units changed from 3:2 to 2:1, showing that BD and AN units were released by cleavage of the main-chain C–C bonds on both sides of the AN unit, and subsequently, new bonds might be generated by radical rearrangement (Fig. S3). In addition, the result of GC–MS analysis revealed the production of 4-cyano-1-cyclohexene in the gas phase after enzymatic degradation of wNBR (Fig. S4A through C). Interestingly, this degradation pattern was identical to that of NBR pyrolysis (6). These findings indicate that certain NBR-degrading enzymes possess the ability to cleave main-chain C–C bonds. The enzymes of the ppt fraction were activated by H_2_O_2_, suggesting that an oxidative degradation was caused through a similar mechanism to a shunt pathway in cytochrome P450. Moreover, oxygen consumption was observed during the enzymatic reaction using the ppt fraction, and aldehyde/ketone was detected by using Schiff’s reagent and the DNPH method.

**TABLE 2:** ^13^C-NMR analysis of wNBR

Sample	Type of carbon (molar ratio)			Acrylonitrile units (wt%)
	C≡N	C=C	CH₂, CH	
Ctrl. 1^*a*^	1.0	1.5	5.6 (calculated value = 5.0)	39
Ctrl. 2^*b*^	1.0	1.6	5.6 (calculated value = 5.2)	38
After enzymatic reaction^*c*^	1.0	1.9	6.5 (calculated value = 5.9)	34

^*a*^ Unused wNBR sample before the enzymatic reaction.

^*b*^ wNBR sample after 24-h incubation with 20 mM Tris-HCl (pH 8.0) at 37°C.

^*c*^ wNBR sample after 24-h incubation with a partially purified enzyme sample under the same conditions as above.

**Fig 4:**
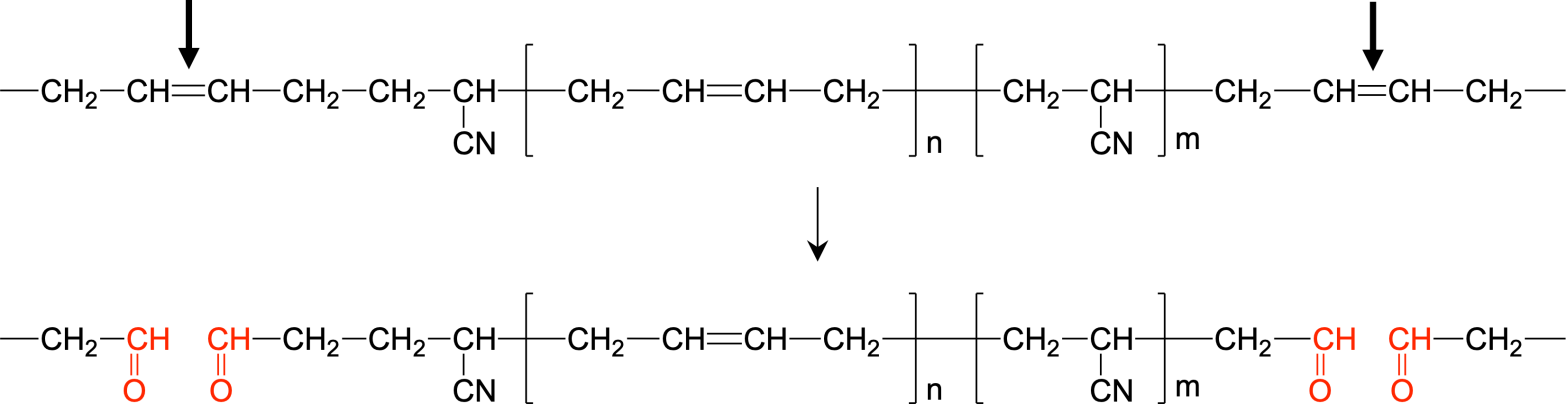
Proposed oxidative cleavage mechanism catalyzed by Nro1.

Besides, the reaction products expected to be the NBR oligomers with *m/z* 680–2,275 in the aqueous phase were detected by MALDI-TOF-MS analysis (Fig. S5A). Among them, based on the *m/z* value of signal no. 1–7, which were estimated to be the degradation products, we thought that the enzymatic reaction products would be the oligomers consisting of BD units (n = 1, 4, 9, 10, 12, 13) and AN units (m = 2, 3, 8–10) with –H, –CH_3_, –OH, –CHO, –COOH groups (Fig. S5B). In particular, signal no. 7 was attributed to an oligomer consisting of BD units (n = 13) and AN units (m = 3), and possibly a dicarboxylate. The formation of the dicarboxylate suggests that the aldehyde groups were generated via cleavage of main-chain C=C bonds and were subsequently oxidized (Fig. 4). These results were in good agreement with the reports of degradation products of IR degradation by known Lcps (3, 4). Such oligomeric products might be useful chemical compounds. Attempts to determine the more detailed chemical structures of the oligomeric products are currently in progress.

As shown in Fig. S6, the white precipitates were produced during the enzymatic degradation of wNBR and pNBR, respectively. For the white precipitates, the result of FT-IR analysis suggested that they may be comprised of an ester with C=C bonds but no CN group (Fig. S6C and D). We are currently investigating the chemical structure of white precipitates to determine whether they were generated by cleavage of main-chain C–C bonds. Moreover, carbon black, as well as white precipitates, were generated in the reaction mixture after the enzymatic reaction. In general, since carbon black constitutes approximately 40 wt% of NBR products, its recycling would be highly desirable.

A whole genome analysis revealed that strain J1A has 3,689 coding sequences, including 59 oxygenase genes containing one alkane 1-monooxygenase (AlkB) gene and 39 oxidase genes. RNA-seq analysis revealed a 7.0-fold increase in the level of transcription of a gene named *nro1* (nitrile rubber oxygenase) compared with the level during cultivation in the presence and absence of wNBR (Table 1). Conversely, the transcriptional level of the gene that encodes AlkB (i.e., *alkB*) decreased to 89% in the presence of wNBR compared with the absence of wNBR. Therefore, we hypothesized that *nro1* is one of the nitrile rubber oxygenases involved in wNBR degradation.

**Fig 5:**
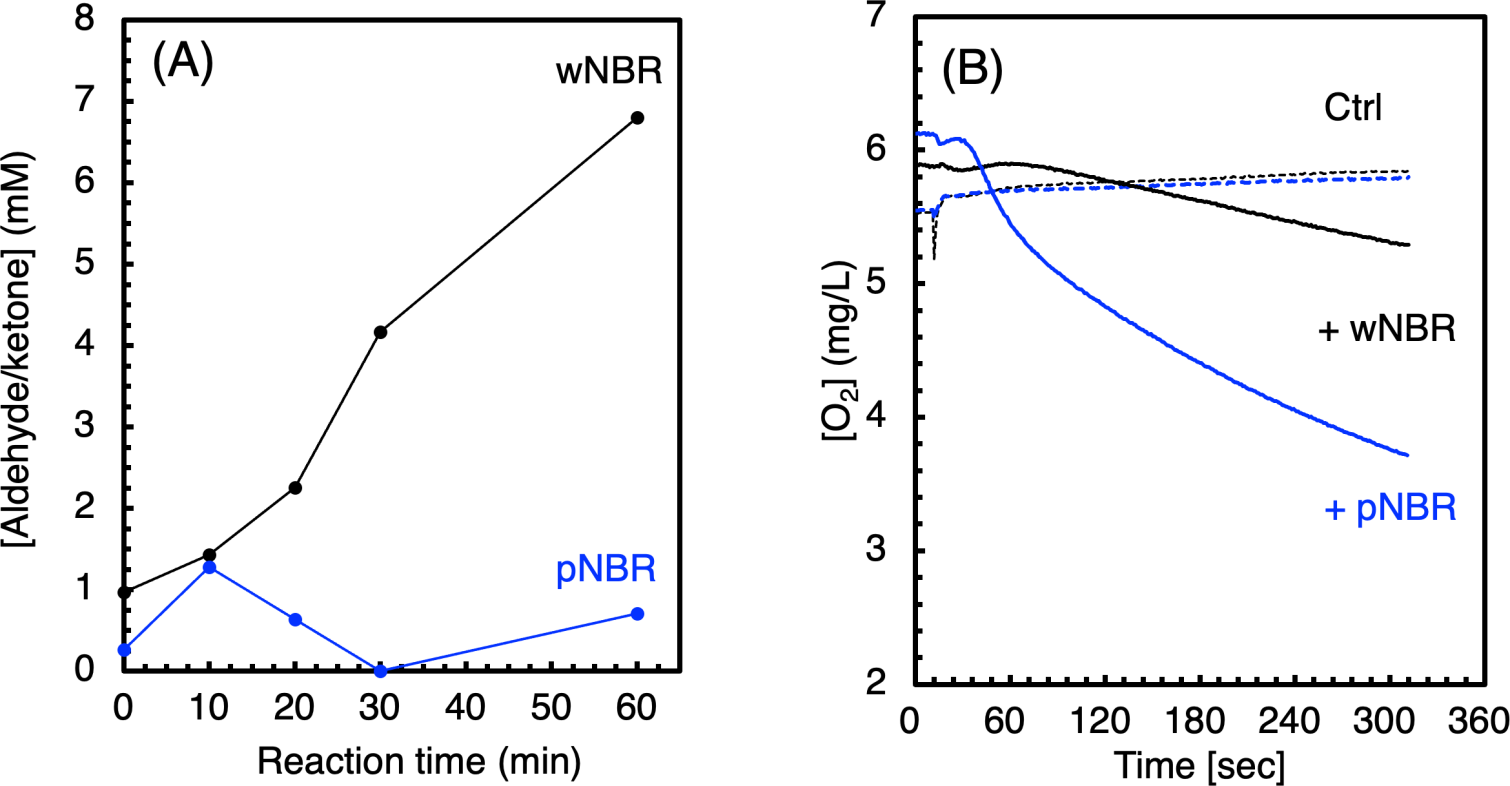
Aldehyde/ketone production (**A**) and O_2_ composition (**B**) in NBR enzymatic degradation by rNro1. (**A**) The cell lysate of the *E. coli* expressed rNro1 was mixed with wNBR (black) or pNBR (blue) and 5 mM H_2_O_2_ and incubated at 37°C. The concentration of aldehydes/ketones in the enzymatic reaction solution was determined by the 2,4-DNPH method. (**B**) Oxygen consumption was measured by an O_2_ monitor during the enzymatic NBR degradation at 37°C over 5 min. Solid and dotted lines represent the enzymatic reaction with lysate and buffer control (Ctrl), respectively.

The recombinant *E. coli* produced approximately 0.1 mg/L-culture of recombinant (rNro1) in the soluble fraction (Fig. S7A). The UV-Vis spectrum of the cfe showed characteristic absorption bands at 425 nm (Soret), 533 nm (β), and 562 nm (α), suggesting that rNro1 carries a b-type heme. (Fig. S7B). The lysate of recombinant cells expressed Nro1, which degraded 0.20% ± 0.03% and 0.07% ± 0.10% weight loss of the wNBR and pNBR samples over 24 h at 37°C, respectively. In addition, net 5.2 mM aldehydes/ketones were detected in the enzymatic degradation of wNBR at 37°C over 60 min (Fig. 5A). In contrast, the production of aldehydes/ketones involved in the enzymatic decomposition of pNBR stopped in 10 min. It is known that purified cytochrome P450 2B4 is inactivated by aldehydes in a reconstituted reaction (7). Therefore, we hypothesized that rNro1 would be inactivated by the aldehydes produced during the enzymatic reaction. We also presumed that the stronger inhibition in the degradation of pNBR compared to that of wNBR may be due to the additives in wNBR scavenging aldehydes. In fact, an anti-aging agent and antioxidant, such as *N*-(1,3-dimethylbutyl)-*N′*-phenyl-1,4-phenylenediamine (6PPD), which carries two amino groups, was added to wNBR. However, in the short-reaction period, the oxygen consumption rate in the degradation of pNBR was faster than that of wNBR (Fig. 5), indicating that the oxidation was proceeding, but the subsequent reaction steps, namely, the steps leading to aldehyde formation, could not proceed. In support of this hypothesis, the weight loss rate due to degradation of pNBR was remarkably lower than that of wNBR as described above. The molecular density of the polymer chain of poly(acrylonitrile-butadiene) of pNBR is higher than that of wNBR because of containing various additives, such as carbon black (ca. 40 wt%), plasticizers, and 6PPD, as well as sc structure. Taken together, we presumed that the polymer chains of pNBR may be less susceptible to continuous attack by rNro1 than those of wNBR, making it less likely that aldehydes will be continuously generated.

The open reading frame encoding *nro1* comprises 882 bp that produces a protein containing 323 amino acids. The deduced molecular weight (33.6 kDa) is very similar to that of the oxygenase MpaB family protein of *Gordonia sihwensis* (MpaB_GS_; GenBank, WP_045537550.1), but lower than that of Lcp 1 from *Gordonia polyisoprenivorans* strain DSM 44266/VH2 (Lcp1_VH2_; GenBank, AFA_75827.1). The deduced amino acid sequence of Nro1 showed 99.3% and 12.8% identities with those of MpaB_GS_ and Lcp1_VH2_, respectively. Though the deduced amino acid sequence of Nro1 showed no similarity with those of known Lcps, the three-dimensional structure of Nro1 was similar to those of Lcps as described below. The MpaB from *Penicillium roqueforti* (MpaB_PR_) is a part of the gene cluster accompanying the biosynthesis of mycophenolic acid and the oxidative cleavage of the C19–C20 double bond in 4-farnesyl-3,5-dihydroxy-6-methylphthalide to yield (4*E*,8*E*)-10-
(4,6-
dihydroxy-
7-
methyl-
3-
oxo-
1,3-
dihydro-
2-
benzofuran-
5-
yl)-4,8-
dimethyldeca-
4,8-
dienoate and acetone via a mycophenolic aldehyde intermediate (8, 9). Reportedly, MpaB_PR_ attacks oxidatively the C=C bond of the terminal dimethylallyl in this reaction pathway. In addition, Purwani et al. reported a new class of bacterial heme-containing oxygenases that can be used for the cleavage of alkene double bonds, analogous to ozonolysis in organic chemistry (2). We therefore propose that Nro1 catalyzes the oxidative cleavage of the main-chain C=C bond in the poly(acrylonitrile-butadiene) component of NBR (Fig. 5).

The predicted structure of Nro1 was similar to the crystal structure of Lcp_K30_ from *Streptomyces* sp. K30 (10). Ilcu et al. reported that Lcp_K30_ has highly conserved amino acid residues (E148*,* R164, K167, T168, and H198) that play important roles in IR catalytic degradation (10). Multiple sequence alignment analysis suggested that R68, K71, and R72 in Nro1 corresponded to R164, K167, and T168 in Lcp_K30_, respectively (Fig. S8A). When the predicted structure of Nro1 was superimposed on the crystal structure of Lcp_K30_, D57, and H104 in Nro1 corresponded spatially to E148 and H198 in Lcp_K30_ (Fig. S8C). Since it has been reported that the K167 and H198 residues in Lcp_K30_ interact with heme (10), we inferred that the corresponding residues in Nro1 (i.e., K71 and H104) may also interact with heme. It has been reported that oxygenases of the MpaB family protein and Lcps carry b-type heme (2, 11). Thus, we postulate that Nro1 is an oxygenase carrying b-type heme.

## Data Availability

The sequence of the genomic DNA of strain J1A is available under NCBI/ENA/DDBJ accession numbers AP041607 for the chromosome and AP041608 for the plasmid. BioSample metadata are available in the NCBI/ENA/DDBJ BioSample database under accession number SAMD01577600. All data are available in the main text or the supplemental material.

## SUPPLEMENTAL MATERIAL

Supplemental figures aem.02128-25-s0001.pdf Fig. S1 to S8.

ASM does not own the copyrights to Supplemental Material that may be linked to, or accessed through, an article. The authors have granted ASM a non-exclusive, world-wide license to publish the Supplemental Material files. Please contact the corresponding author directly for reuse.
