# Supplementary material for: Nitrile rubber biodegradation by Gordonia sp. strain J1A and discovery of an oxygenase involved in its degradation
Source: Appl Environ Microbiol. 2025 Nov 26;91(12):e02128-25. doi: 10.1128/aem.02128-25 (PMC12724181; doi:10.1128/aem.02128-25)
Supplement: Supplemental figures — Fig. S1 to S8. [file aem.02128-25-s0001.pdf]

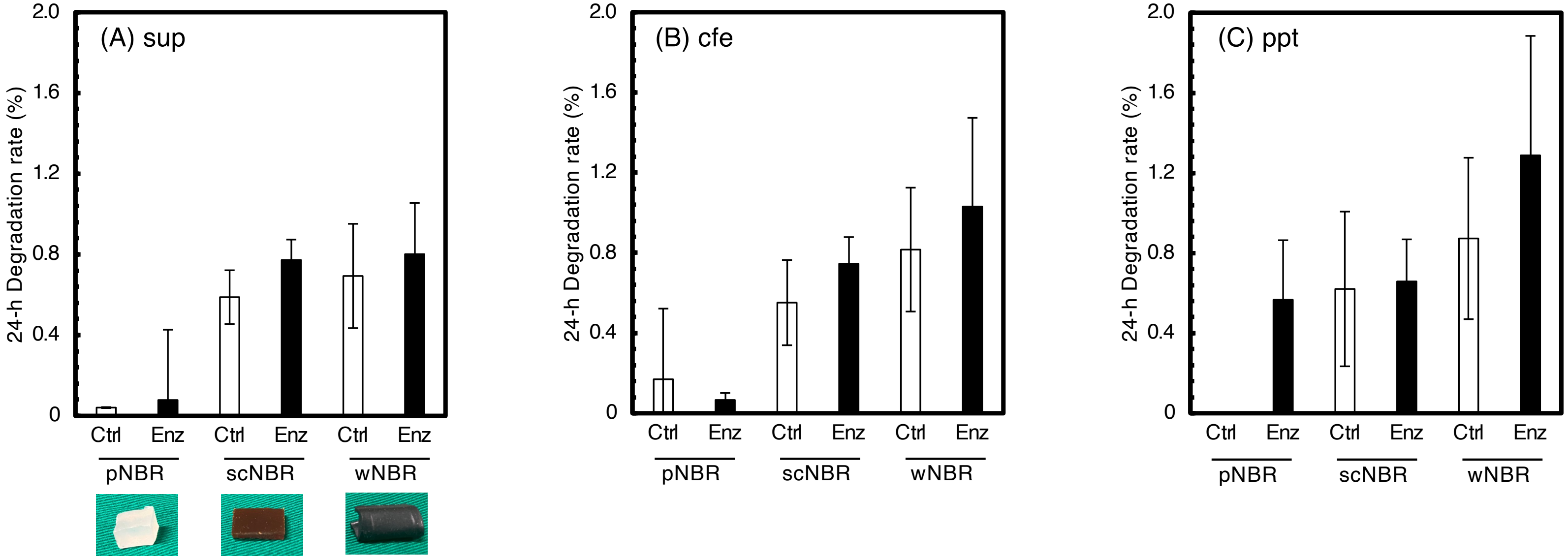

Fig. S1. Degradation of each NBR sample by each cellular fraction containing NBR-degrading enzymes.
The degradation rate was determined based on the weight loss after 24-h incubation at 37ºC in 20 mM Tris-HCl buffer (pH 8.0). As the tested NBR sample, wNBR, scNBR and pNBR.

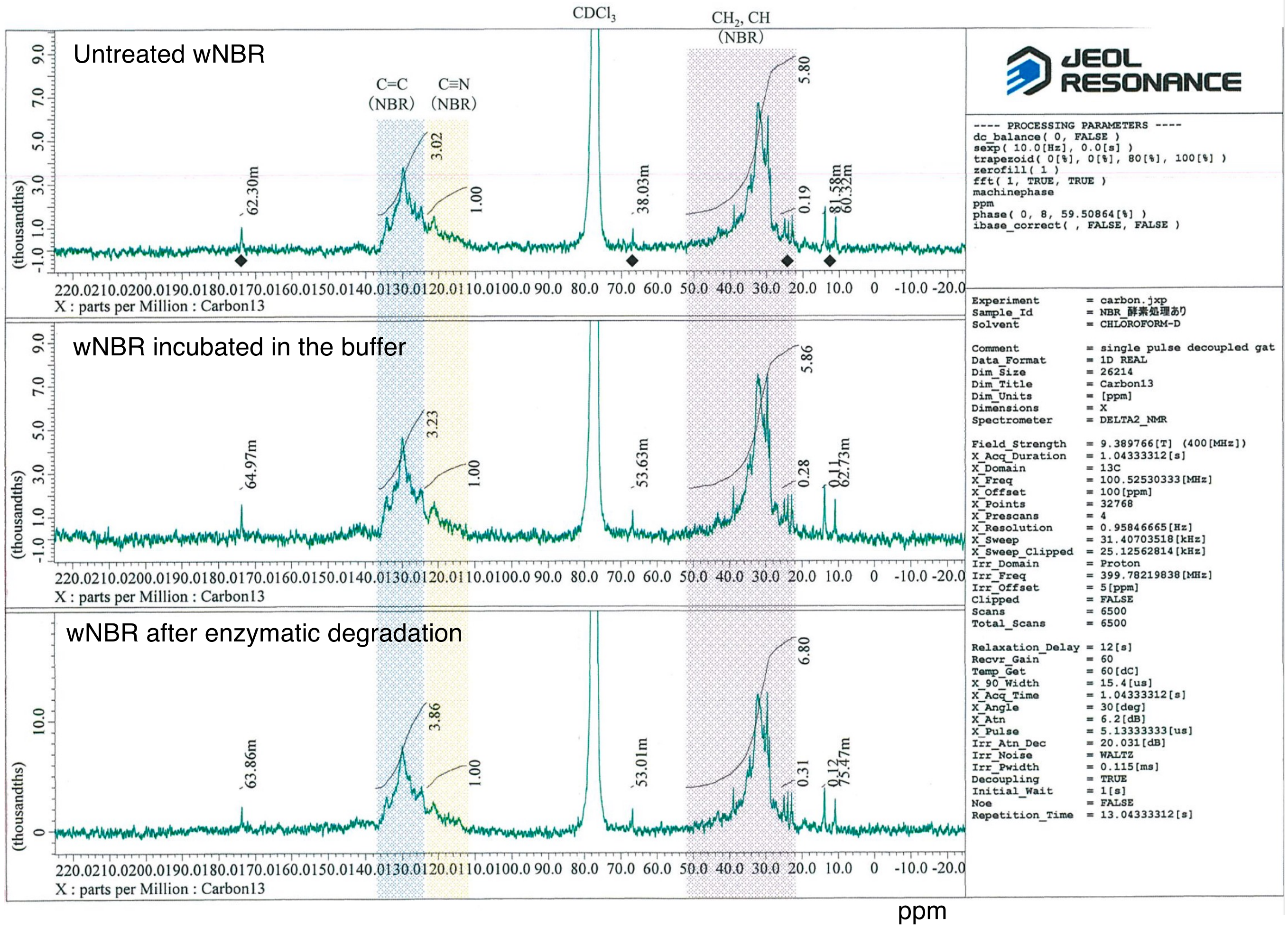

Fig. S2. ^{13}C-NMR spectra of wNBR sample of pre- and post-enzyme reaction.

^{13}C-NMR spectroscopic analysis was done by using $CDCl_3$ as the solvent at 60ºC. The active fraction of the anion exchange (DEAE) column chromatography was used to the enzymatic wNBR degradation. ◆, peaks from additives in a vial septum.

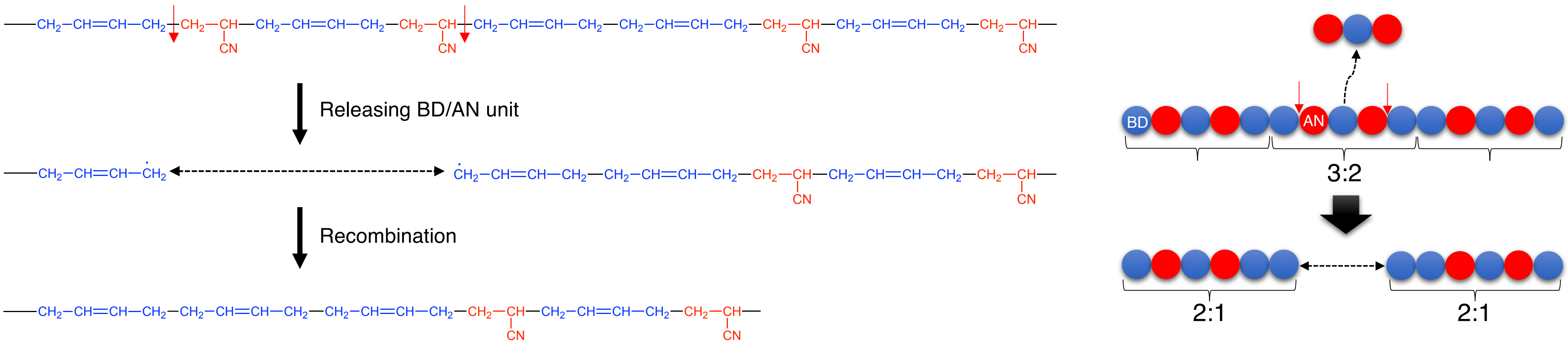

Fig. S3. Proposed degradation pattern based on ^{13}C-NMR analysis.

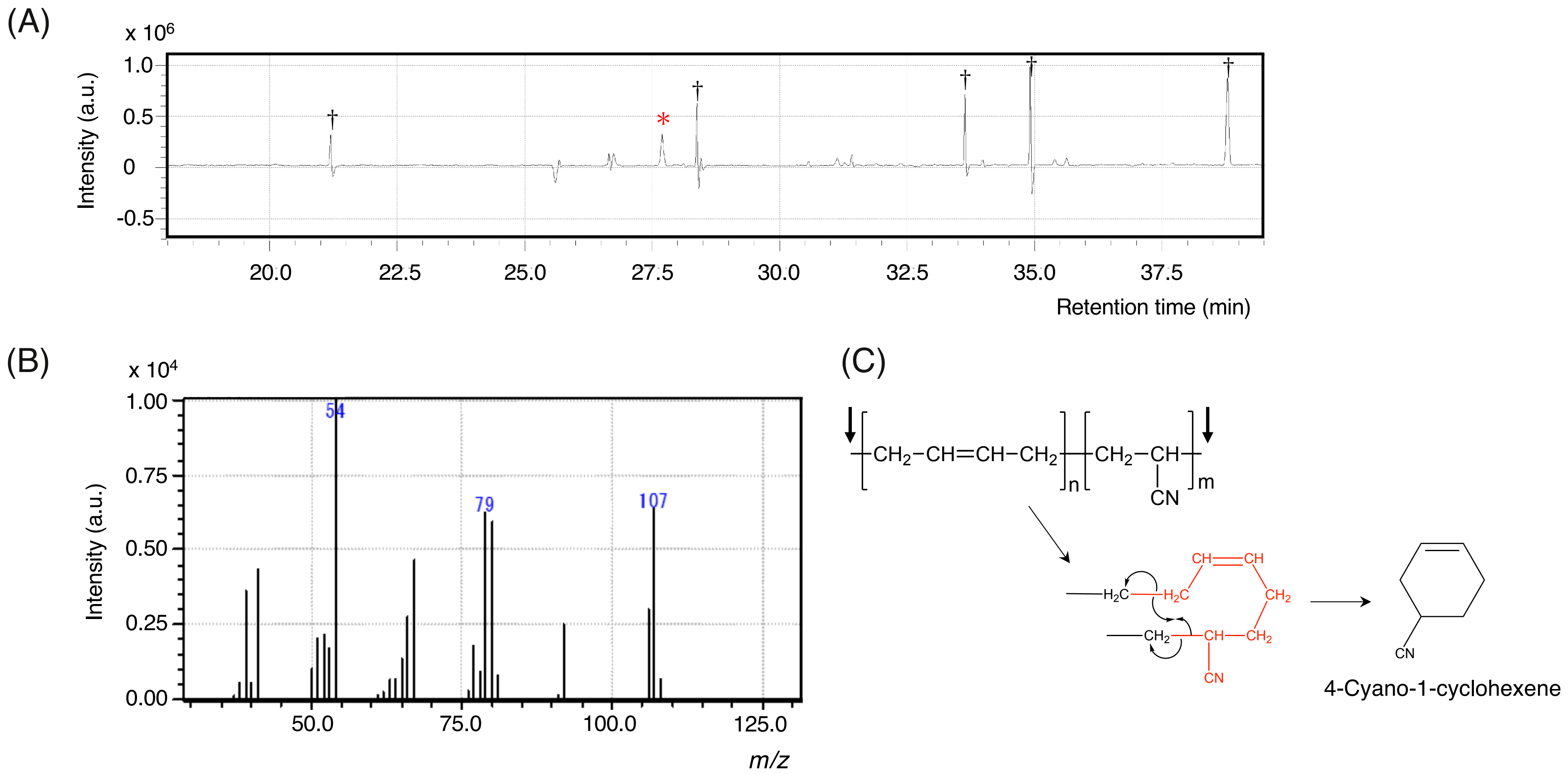

Fig. S4. GC-MS analysis of the gas phase after the enzymatic degradation of pNBR.
(A, B) The gas phase in the head space after the 24-h enzymatic reaction with pNBR and the cellular precipitate fraction at 37ºC was analyzed by GC-MS. *, predicted enzymatic degradation product; †, peaks from the sampling vessel, cap, and column. (C) The bonds cleaving by the enzymatic reaction that predicted based on the reaction product, 4-cyano-1-cyclohexene detected in the gas phase.

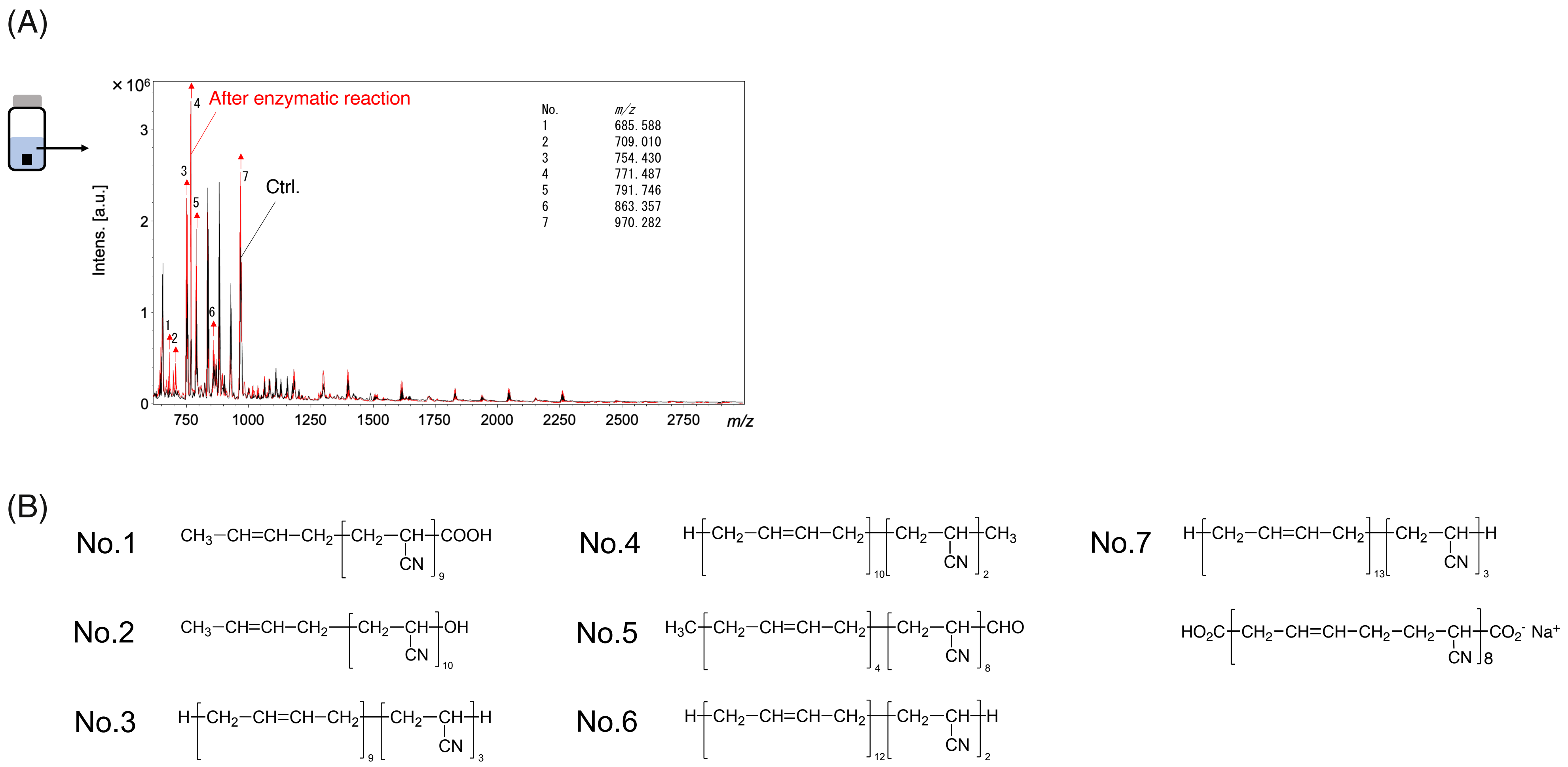

Fig. S5. MALDI-TOF-MS analysis of the aqueous sample after the enzymatic reaction.

(A) The enzymatic reaction with wNBR and the cellular precipitate fraction was carried out for 24 h at 37ºC and pH 8.0. Black and red line are the spectra of the buffer control and the aqueous sample after the enzymatic reaction, respectively. (B) Examples of the chemical structures of the reaction products postulated based on the *m/z* value.

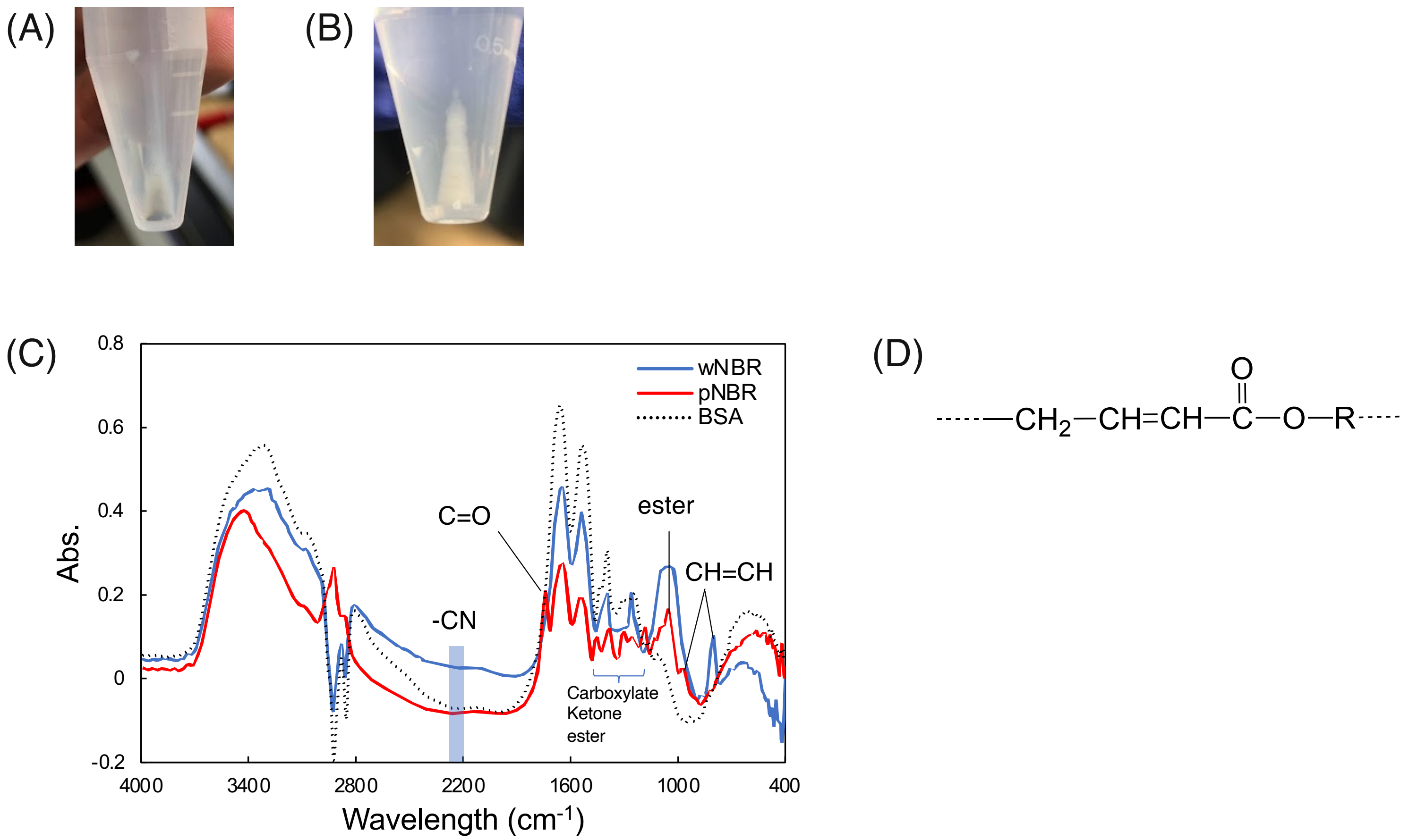

Fig. S6. FT-IR analysis of the precipitates produced by the enzymatic degradation of pNBR.

(A) Precipitates generated by the enzymatic degradation of wNBR. (B) Precipitates produced by the enzymatic degradation of pNBR. The enzymatic reactions with wNBR and pNBR were carried out using the partially purified enzyme (DEAE active fraction) for 24 h at 37ºC and pH 8.0. (C) FT-IR spectra of bovine serum albumin (control, dotted line), the enzymatic degradation product from wNBR (blue), and that from pNBR (red). (D) the predicted chemical structure of the enzymatic degradation product.

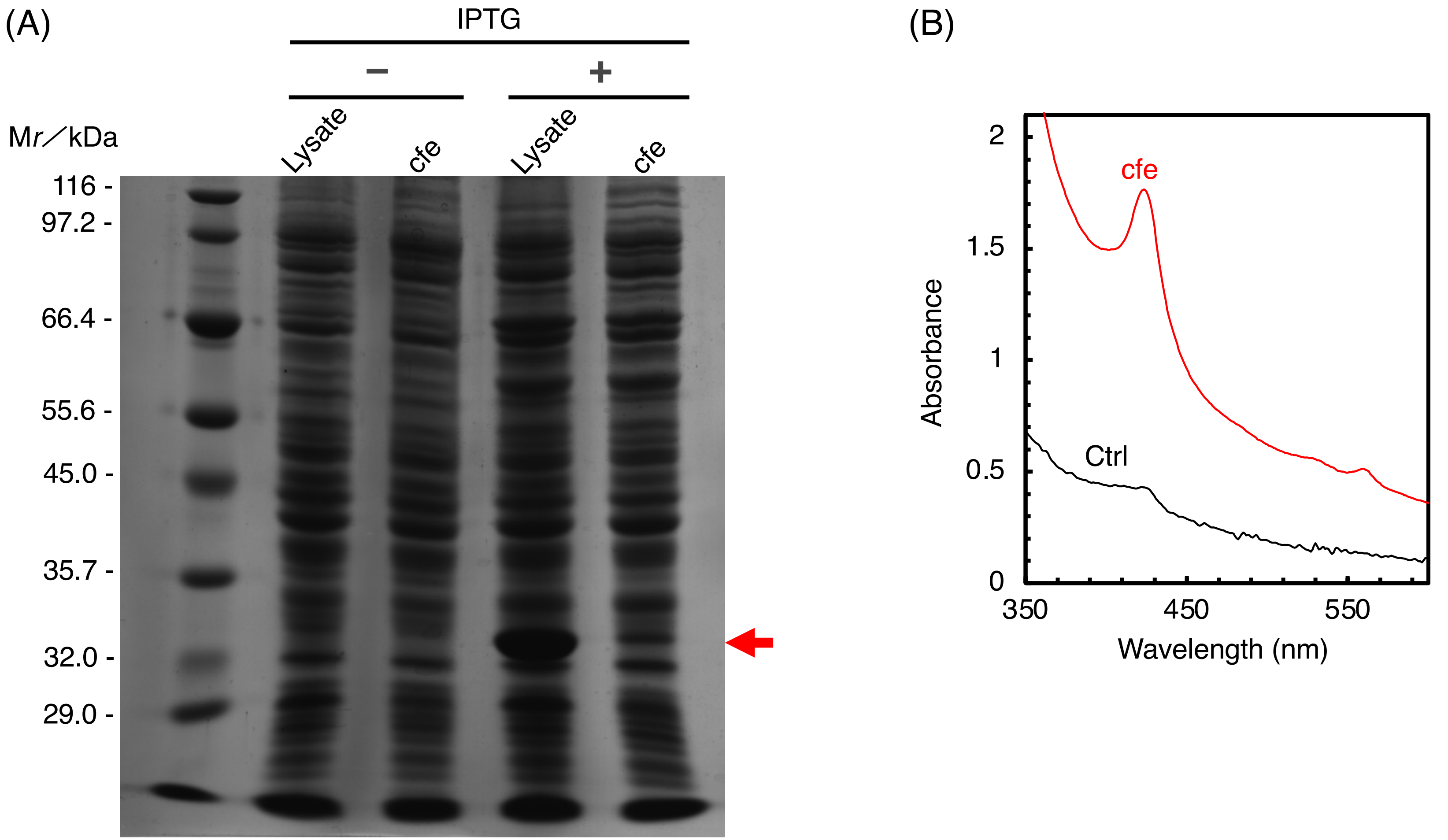

Fig. S7. SDS-PAGE analysis (A) of rNro1 recombinant expression by *E. coli* cells and UVvis spectrum (B) of cfe containing rNro1.
(A) 10%-running gel stained by CBB. Lysate or cfe for 30 μL of culture medium was loaded onto the gel. rNro1 was expressed in *E. coli* strain C43 (DE3) by 4-h IPTG induction at 30ºC. (B) the cfe of the recombinant cells (red) and cfe of control cells harboring empty vector (black).

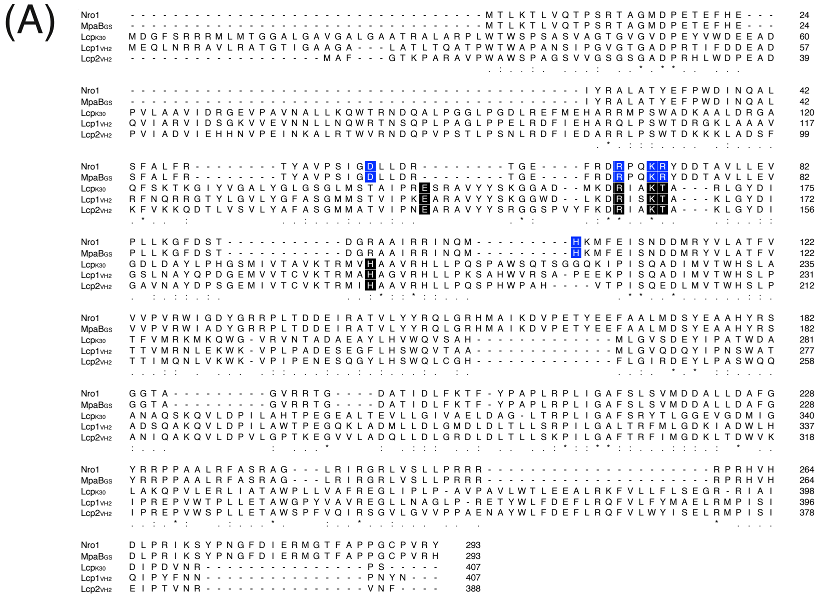

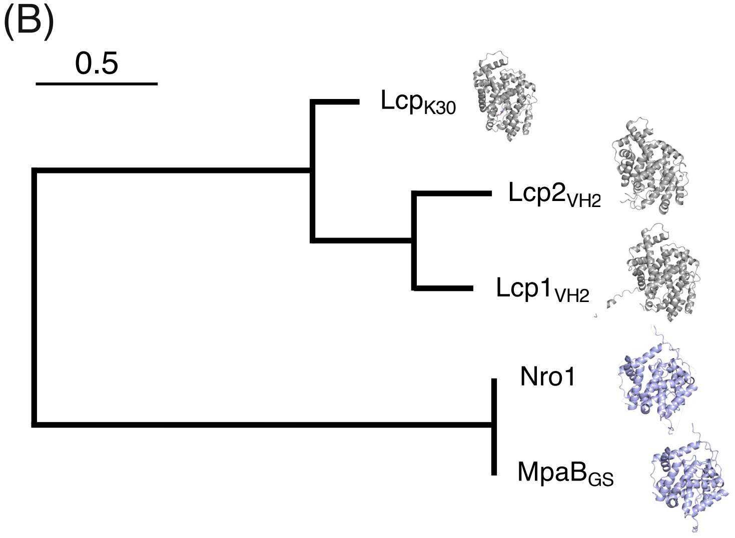

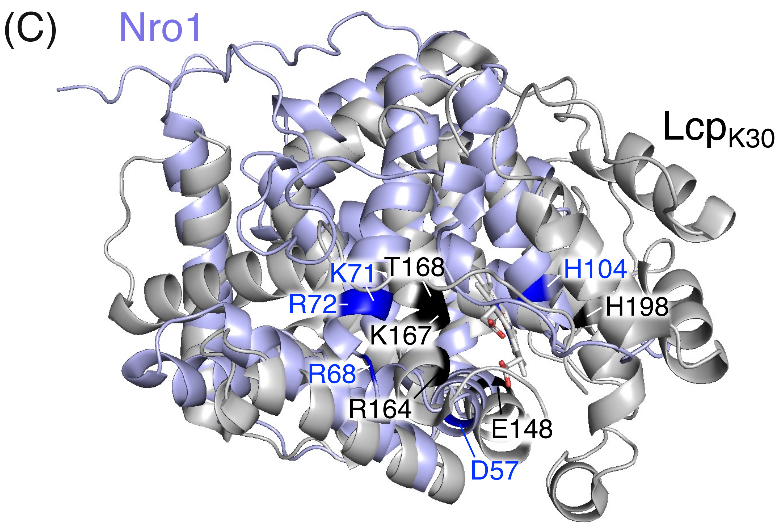

Fig. S8. Multiple sequence alignment (A) and phylogenetic tree (B) of mpaB family proteins and structure superimposition between Nro1 and Lcp$_{K30}$ (C).

(A, B) The multiple sequence alignment (MSA) and phylogenetic tree were constructed for Nro1, MpaB$_{GS}$ (WP_045537550.1), Lcp$_{K30}$ (Q3L8N0.2), Lcp1VH2 (AFA75827.1), and Lcp2$_{VH2}$ (AFA76036.1) using CLUSTALW (https://www.genome.jp/tools-bin/clustalw; accessed on 8/10/2023) and ETE3 (https://www.genome.jp/tools-bin/ete; accessed on 11/08/2023), respectively. In the MSA, the highly conserved amino acid residues (E148, R164, K167, T168, and H198 in Lcp$_{K30}$) that play important roles in the catalytic reaction are highlighted in black. The blue highlighted amino acid residues (D57, R68, K71, R72, and H104) in Nro1 corresponded with the the important amino acid residues of Lcp$_{K30}$. (B) In the phylogenetic tree, the scale bar corresponds to a genetic distance. The crystal structure model of Lcp$_{K30}$ (Protein Data Bank ID: 5O1M) and the structural models of Nro1, Lcp1$_{VH2}$, and Lcp2$_{VH2}$ predicted by ColabFold (https://colab.research.google.com/github/sokrypton/ColabFold/blob/main/AlphaFold2.ipynb; accessed on 8/10/2023) is depicted. (C) The structures of Lcp$_{K30}$ and Nro1 were superimposed by Pymol Molecular Graphic System Version 2.5.2.
